# Coping during wartime: a qualitative study on secular existential coping strategies of Ukrainians

**DOI:** 10.3389/fsoc.2026.1889407

**Published:** 2026-10-02

**Authors:** Nataliia Melnyk, Fereshteh Ahmadi, Olena Kovtun

**Affiliations:** 1 National University “Kyiv Aviation Institute”, Kyiv, Ukraine; 2 University of Gävle, Gävle, Sweden

**Keywords:** wartime coping strategies, existential coping, secular meaning-making, Ukrainian civilians, qualitative study

## Abstract

**Introduction:**

Russia’s full-scale invasion of Ukraine has generated profound psychological and existential challenges. Although religious coping has been widely studied, secular existential meaning-making during wartime remains underexplored.

**Methods:**

A qualitative design was used. Semi-structured interviews were conducted with 67 adults aged 20-72 years from diverse occupational, regional, and religious backgrounds. Data were analysed using reflexive thematic analysis.

**Results:**

Five interrelated themes were identified: nature and physical space as sources of existential grounding; cultural and creative practices; civic engagement and solidarity; routine, work, and practical activity; and physical activity, meditation, and non-religious contemplative practices. These strategies were embedded in cultural heritage, collective historical memory, personal agency, and social connection.

**Discussion:**

Secular existential meaning-making is a culturally grounded pathway of adaptation that can complement religious and clinical models of support. Psychosocial interventions in conflict settings should recognize psychological, existential, and cultural resources without treating religious identity as determinative of the coping strategies used.

## Introduction

In the context of contemporary global instability, wars, armed conflicts, natural disasters, and socio-political crises are occurring with increasing frequency, deeply affecting individuals, communities, and entire societies. The psychological impact of such traumatic events is particularly acute in regions experiencing prolonged violence and instability. Eriksen describes modern society as the “century of trauma” (Eriksen, 2014), as trauma permeates the discourse surrounding nearly all major adversities worldwide. Studies have shown that up to 60% of Americans experience at least one traumatic event in their lifetime, while this figure rises to 92% in conflict-affected regions such as Iraq, Algeria, and Cambodia (Friedman, 2016). The ongoing war in Gaza, for example, has resulted in widespread destruction, displacement, and severe mental-health consequences. Trauma-related disorders such as PTSD, depression, and anxiety have been reported at high rates among Palestinians, especially among children and displaced persons (Thabet and Vostanis, 2000; War Child, 2024).

Ukraine is currently experiencing one of the most severe humanitarian crises in its modern history. Since the beginning of the full-scale Russian invasion on 24 February 2022, Ukrainians have faced displacement, loss of loved ones and homes, continuous threats to their safety, and the destruction of critical infrastructure. In 2022, Ukraine’s Minister of Health, Viktor Liashko, stated that approximately 15 million Ukrainians would require psychological support, with up to 4 million needing medical treatment for mental health disorders (Liashko, 2022). According to WHO estimates, one in five individuals living in a war zone is likely to experience moderate to severe mental health problems (World Health Organization, 2022). These circumstances have led to widespread psychosocial stress and trauma across all segments of the population, including civilians, internally displaced persons (IDPs), and military personnel (Rybinska et al., 2023). Recent analyses indicate that more than 15 million Ukrainians have reported deterioration in their mental health since the start of the war as a result of bereavement, displacement, instability, and other stressors (Nguyen, 2022; International Organization for Migration, 2023). Yurtsenyuk and Sumariuk (2023) describe war as a powerful psychosocial stressor contributing to neurotic and stress-related disorders and estimate that depression, anxiety, and PTSD may affect almost 9.6 million Ukrainians. More recent monitoring indicates that 84% of Ukrainians report mental health problems (Liashko, 2025).

While clinical responses to trauma, such as psychiatric care and treatment for PTSD, remain essential, there is growing recognition of the role played by coping strategies, particularly those related to meaning-making and existential adaptation. Foundational research on stress and coping (Lazarus and Folkman, 1984; Compas et al., 2001) highlights the importance of understanding both problem-focused and emotion-focused coping mechanisms used by individuals in extreme psychological circumstances. In wartime contexts, where existential uncertainty and loss are dominant features of daily life, secular coping strategies encompassing personal belief systems, philosophical reflection, and internal resources may play a crucial role in supporting psychological resilience.

Although research on wartime coping has expanded in recent decades, including studies of civilians under the Gaza blockade (Hammad and Tribe, 2021) and Yugoslav citizens during aerial bombardment (Pejičić et al., 2018), the Ukrainian case requires further study as the war continues and the social context changes from month to month. Existing studies have focused on coping among IDPs during the 2014 conflict (Bohucharova and Tkachenko, 2016), PTSD and coping among veterans (Turetska and Shtoltsel, 2016), and the psychological responses of civilians in the early days of the 2022 invasion (Khraban, 2022).

Previous studies have addressed coping and resilience from different angles. Some have examined the observable and measurable aspects of how individuals and groups manage crises (Ahmadi, 2006; Folkman and Moskowitz, 2004), while others have explored how broader contextual factors—such as war, social disruption, or collective trauma—shape people’s capacity for resilience (Pomytkina et al., 2023; Melnyk, 2022). This variety of perspectives suggests that coping and resilience should be studied not only as psychological mechanisms but also as socially and culturally embedded phenomena, requiring an integrated approach that links psychological research with sociocultural and existential analyses within the humanities.

However, secular existential coping strategies—non-religious yet deeply personal and culturally embedded ways of making sense of trauma—remain underexplored, especially in cultural contexts where religion has traditionally played a central role, such as Ukrainian society. This research seeks to address that gap by examining how Ukrainians have coped with adversity during the full-scale war, with a specific focus on secular existential coping. These strategies include the search for meaning through nature, art, interpersonal relationships, civic solidarity, and engagement with cultural identity. Such practices may offer alternative or complementary pathways to resilience, alongside religious, clinical, or institutional forms of support.

### Theoretical framework

The formation of coping strategies has acquired new significance, both individually and collectively, in the context of Russia’s full-scale invasion of Ukraine (Sodolevska, 2024). Recent Ukrainian studies confirm that the war has posed an acute challenge to the mental health of the population, particularly among young people, who have had to rapidly adapt to new and often traumatic realities. This adaptation has required the mobilization of internal resources and the use of both traditional and emerging coping strategies, many of which are secular and non-religious in nature (United Nations Development Programme, 2024, 2025). Among these strategies, researchers have noted a growing orientation toward creativity, civic engagement, self-reflection, mutual support, and re-evaluation of personal meaning (International Medical Corps, 2024). In this context, active and positive coping strategies—including volunteering, acts of solidarity, and the maintenance of daily routines—have proven effective in supporting emotional wellbeing and forming the basis for psychological recovery in prolonged crisis settings (Korniievskyi, 2015).

Lahad’s BASIC Ph model conceptualizes coping and resilience as drawing on multiple channels and has informed community-resilience programmes (Lahad, 2016). In research with Ukrainian youth, Giordano et al. (2024) found that adaptive resources such as positive thinking, perceived control, emotional awareness and regulation, close relationships, and community support were associated with well-being and self-confidence. These findings highlight the contribution of cognitive, behavioural, emotional, social, and culturally situated resources to resilience during wartime.

In a broader European context, empirical studies among adolescents and young adults suggest that religious and secular coping strategies are often intertwined. Rather than being mutually exclusive, they frequently co-exist within individual meaning-making processes. Within these practices, personal narratives, belief systems, and cultural-symbolic resources play a central role—independent of institutionalized religion (Zandi and Ahmadi, 2024).

Several European studies have shown that a coherent secular worldview, when supported by cultural institutions and social networks, correlates positively with psychological wellbeing (Wnuk, 2024). Publications under the “Secularity and Wellbeing” initiative by Oxford University Press argue that individuals can adapt effectively to adversity by drawing on internal values, philosophical reflection, and relational bonds, even in the absence of religious belief (Sisto et al., 2019).

Folkman and Moskowitz (2004), working within the cognitive-behavioural tradition, identified key coping mechanisms used by individuals in situations of acute or chronic stress. These include:

- Problem-focused coping (e.g., planning, goal-setting, active problem-solving);
- Emotion-focused coping (e.g., emotional expression, withdrawal, self-soothing);
- Positive reappraisal (e.g., finding meaning in adversity through reflection, values, or support).

They also emphasised the essential role of social relationships—family, peers, and professional communities as a resource in stress mitigation. Drawing on the foundational work of Lazarus and Folkman (1984), their approach highlights the dynamic nature of coping: it is not a fixed trait, but a set of adaptive strategies shaped by context, experience, and access to internal and external resources.

### Secular existential meaning-making coping

Secular existential meaning-making coping refers to the ways individuals manage profound existential concerns without relying on religious or spiritual belief systems. This form of coping has become increasingly relevant in modern secular societies, where traditional religious frameworks no longer serve as the primary source of existential support. Instead, individuals turn to alternative, non-religious resources to construct meaning and maintain psychological wellbeing when facing life’s most profound challenges.

Ahmadi and her colleagues (Ahmadi et al., 2018; Ahmadi et al., 2022; Ahmadi et al., 2024; Zandi and Ahmadi, 2024; Zandi et al., 2023) explored coping strategies used by individuals facing serious life adversities across ten countries, and identified three main categories: religious coping (e.g., prayer, turning to God, participation in religious communities); spiritual coping (e.g., thinking about a spiritual force, experiencing a strong spiritual feeling, getting a spiritual feeling by helping others, listening to spiritual music, feeling a strong spiritual connection with other people, trying not to think about one’s illness by pondering spiritual issues, hoping for a spiritual rebirth in this world); and secular existential meaning-making (e.g., existential connection with oneself, sanctification of nature, positive solitude, empathy/altruism, reflections on the meaning of life). Special emphasis was placed on the cultural contexts in which these strategies emerged. Ahmadi introduced the concept of “meaning-making coping.” Meaning-making coping refers to the process through which individuals attempt to understand and manage major life challenges—such as illness, loss, or crisis—by searching for meaning and significance. It encompasses a wide range of strategies that may be *religious, spiritual, or secular existential* in nature. These coping methods help people restore a sense of coherence and purpose when their existing worldview is disrupted. In this approach, meaning-making coping is viewed as culturally and historically embedded, shaped by individuals’ socialisation and the cultural context in which they live (Ahmadi and Ahmadi, 2018). Their findings highlighted the profound influence of cultural narratives and worldviews on how individuals cope with trauma. In the present study, religious, spiritual, and secular existential coping were treated as analytically distinct but empirically permeable categories. Religious coping was defined as meaning-making explicitly oriented toward God, prayer, worship, religious doctrine, or participation in a religious institution. Spiritual coping referred to appeals to a transcendent force, universal energy, sacred interconnectedness, or inner spirituality not mediated by organized religion. Secular existential coping referred to the construction of meaning, coherence, agency, or belonging through non-transcendent resources, including nature, creative practice, work, civic solidarity, personal values, reflection, and embodied routines. Importantly, classification concerned the coping resource described in a particular interview segment rather than the participant’s declared religious identity. Thus, a participant who identified as religious could nevertheless describe a secular coping strategy. Where secular and religious or spiritual resources co-occurred in the same narrative, the account was classified as hybrid, and only the analytically distinguishable secular component was included in the development of the present themes.

Although some Ukrainian scholars have acknowledged the role of secular, non-religious approaches to meaning-making in supporting mental wellbeing, these coping strategies have yet to be systematically studied in practice—a gap this research aims to address.

Our study also draws on resilience theory, which emphasizes the capacity of individuals and communities to adapt to and recover from extreme adversity (Masten, 2014). In the context of wartime Ukraine, resilience is shaped not only by individual coping strategies and social support networks but also by collective memory and national identity (Hornostai, 2024). Ukraine’s history of repeated large-scale trauma, including the Holodomor, Soviet repression, and the Chernobyl disaster, has contributed to the formation of a cultural foundation for endurance and meaning-making (Ohiienko, 2018; Hornostai, 2024). These historical experiences have fostered adaptive mechanisms such as civic engagement, cultural expression, and solidarity-based mutual aid. Empirical studies suggest that narratives of historical resistance and perseverance serve as psychological resources, enabling individuals to navigate trauma, affirm identity, and maintain emotional stability during periods of crisis (Hirschberger, 2018). This culturally embedded resilience underscores the importance of context-sensitive mental health interventions in conflict-affected regions of Ukraine.

## Methodology

This study was conducted as part of a larger international research project on meaning-making coping, carried out through a collaboration between scholars from the University of Gävle (Sweden) and the State University “Kyiv Aviation Institute” (Ukraine). The study is based on a qualitative approach that allows for a deep analysis of the individual experience of Ukrainians in the context of existential coping during the war, focusing primarily on secular (non-religious) strategies of comprehension and adaptation. Given the subjective, personal, and culturally embedded nature of these experiences, a qualitative methodology was appropriate for identifying meaning-laden life narratives and processes through which participants built psychological resilience under traumatic conditions.

The first article, entitled “Culture and religion meaning-making coping strategies in Wartime: insights from a qualitative study on the coping experiences of Ukrainians,” addressed how Ukrainians use religious and spiritual meaning-making coping strategies during the ongoing war, and how these strategies are shaped by cultural, historical, and personal factors. The present article is the second in this series and focuses specifically on secular existential meaning-making coping strategies, thereby examining adaptive mechanisms and meaning-construction processes that operate independently of religious or institutional frameworks.

This thematic concentration allows for a more rigorous and granular analysis of existential coping narratives, particularly those rooted in secular philosophical outlooks, humanistic values, and culturally specific interpretations of life under extreme adversity. This empirical investigation constitutes the second publication derived from an extensive qualitative research project on coping strategies among Ukrainians during wartime. Given the substantial breadth and depth of the collected empirical material comprising 67 semi-structured, in-depth interviews—its integration into a single publication would have resulted in a significant cognitive overload for the reader and an inevitable compromise in analytic precision. Accordingly, the research team elected to divide the findings into two interrelated but distinct scholarly articles.

The decision to divide the dataset was also methodologically justified by the thematic heterogeneity of the material across gender, age, region, occupation, religious orientation, and life and work experience. These variations required a more nuanced analytical approach. Examining the secular-existential dimension separately ensured a clearer conceptual focus, maintained the coherence of thematic categories, and allowed a more systematic presentation of the findings.

Inclusion criteria were age 20-72 years, residence in Ukraine during the ongoing war, and direct or indirect exposure to the consequences of the war. Exclusion criteria were inability to provide informed consent or a high level of current psychological distress. Participants were recruited through university mailing lists, professional and community networks, non-governmental organizations, and targeted social-media announcements. Limited snowball sampling was used to reach underrepresented groups, including internally displaced persons and religious minorities.

The study employed semi-structured interviews within a qualitative framework to enable open-ended exploration of participants’ lived experiences, coping processes, and interpretations of meaning under extreme adversity. Sixty-seven participants represented diverse regions, occupations, age groups (20-72 years), and religious orientations. The semi-structured protocol was administered primarily through an online survey service (75%); 20% of interviews were conducted face to face and 5% via Zoom or Google Meet, depending on participant preference and connectivity. Data were collected predominantly in Ukrainian. Interviews lasted 25-60 min, typically approximately 40 min.

The interviews were conducted in Ukrainian, either in person or online, depending on the safety conditions and logistical possibilities. The procedure of recruitment and data collection included semi-structured interviews, which were conducted primarily online survey service, 20% of it were face to face, 5% via Zoom or Google Meet, depending on participant preference and connectivity. Interviews were held predominantly in Ukrainian and lasted between 25 and 60 min (sometimes ≈ 40 min). The interview guide had been piloted with five respondents to ensure clarity and relevance. All interviews were translated into English, with bilingual researchers cross-checking for conceptual accuracy. ID numbers were used to preserve anonymity. To ensure the rigor and trustworthiness of the findings, Lincoln and Guba’s (1985) evaluative criteria, as discussed in detail by Loh (2013), were applied. Credibility was strengthened through analyst triangulation. Two researchers independently coded a subset of the interview transcripts and subsequently compared code labels, category boundaries, and interpretations of participants’ meaning-making. Differences were addressed through repeated examination of the complete interview extracts and discussion within the research team. The purpose of this procedure was not to calculate numerical inter-coder agreement, but to challenge individual assumptions and broaden the interpretive perspective.

Candidate themes and their supporting extracts were subsequently reviewed by all authors. During this process, the researchers considered whether each theme was internally coherent, sufficiently distinct from the other themes, and supported by multiple accounts. Coding decisions, revisions of theme definitions, and reflections on the researchers’ cultural and academic positioning were documented in an audit trail and reflexive memos. The original Ukrainian transcripts and their English translations were also repeatedly compared when culturally specific expressions or concepts could not be translated directly. Deviant, ambiguous, and hybrid cases were retained and examined rather than removed from the dataset. Transferability was supported through contextualized descriptions of participants’ socio-demographic, regional, occupational, and religious backgrounds, while dependability and confirmability were strengthened through the documentation and collective review of analytical decisions.

Data collection and analysis proceeded iteratively, with continuous monitoring of emerging codes and themes. After approximately 60 interviews, no substantially new thematic patterns were identified, suggesting that conceptual saturation had been reached. Data collection nevertheless continued to 67 participants to broaden the diversity of experiences represented and to examine potentially disconfirming or underrepresented cases. These strategies collectively strengthened the trustworthiness and interpretive validity of the study.

The analysis followed Braun and Clarke’s (2006) six recursive phases of reflexive thematic analysis: familiarization with the data, generation of initial codes, construction of candidate themes, review of themes, definition and naming of themes, and production of the analytical report. The analysis was predominantly inductive. The researchers first read and reread the interview transcripts and generated data-driven semantic and latent codes without assigning responses to predetermined thematic categories. Related codes were then grouped into candidate themes reflecting recurrent patterns of meaning across the interviews. At the focused coding stage, these candidate themes were examined in relation to the study’s conceptual distinction between religious, spiritual, and secular existential coping. Codes were reviewed against both the relevant interview extracts and the dataset as a whole; overlapping themes were merged, internally heterogeneous themes were divided, and theme boundaries were refined until each theme demonstrated sufficient internal coherence and analytical distinctiveness.

Coping practices were classified according to the meaning participants attributed to them rather than according to the practice alone. For example, volunteering was coded as secular when it was described in terms of responsibility, usefulness, solidarity, agency, or social belonging, but as religious when it was explicitly framed as service to God or obedience to a religious calling. Similarly, meditation or engagement with nature was coded as secular when it was associated with bodily regulation, reflection, or emotional grounding, and as spiritual when participants attributed its effectiveness to a transcendent force or sacred connection. Hybrid coding was used when both types of meaning were explicitly present. Purely religious or spiritual segments were not used to construct the themes reported in the present article.

Participant characteristics are summarized in Figure 1. Women comprised 71.6% of the sample and men 28.4%. Participants aged 35-49 constituted the largest group (64.2%), followed by those aged 50 years or older (19.4%) and those aged 20-34 (16.4%). Regarding regional or displacement status, 40% were classified as Central Ukraine, 24% as Western Ukraine, 16% as Eastern Ukraine, 12% as Southern Ukraine, and 8% as internally displaced persons. In terms of religious affiliation, 44.8% identified as Orthodox Christian (UOC/OCU), 12% as Greek Catholic, 7.5% as Protestant, 4.5% as members of other Christian denominations, 24% as non-religious/secular, and 7.5% as spiritual but not religious. This diversity supported analysis of coping across different demographic and belief backgrounds. Thus, the demographic data we can present in Figure 1, according to:

- *Gender profile* was presented mostly by women, what is explained by the war conditions and among civilians there were women who is more interested in the including to the interviews, thus women comprised 71.6% of the sample, reflecting their strong presence in education, healthcare, and social care-sectors significantly impacted by the war and central to coping-related research. Men, representing 28.4% of participants, were primarily engaged in sectors such as security, infrastructure, and technical professions, providing perspectives shaped by different types of wartime exposure and occupational demands.
- *Age profile showed*, that most participants (64.2%) were aged 35–50, suggesting that the findings mainly reflect the experiences of middle-aged adults, many of whom were combining family and work responsibilities under crisis conditions. Younger adults (25–34) represented 16.4%, providing insights from early-career stages, while older adults (50+) made up 19.4%, contributing perspectives shaped by greater life experience.
- *Regional representations* were drawn from across Ukraine, with the largest share from Central Ukraine (40%), followed by Western (24%), Eastern (16%), and Southern regions (12%). Internally displaced persons (IDPs) constituted 8%.
- *Religious & spiritual diversity* reflect Ukraine’s diverse spiritual landscape, the largest group identified as Orthodox Christian (UOC/OCU) at 44.8%.

**Figure 1:**
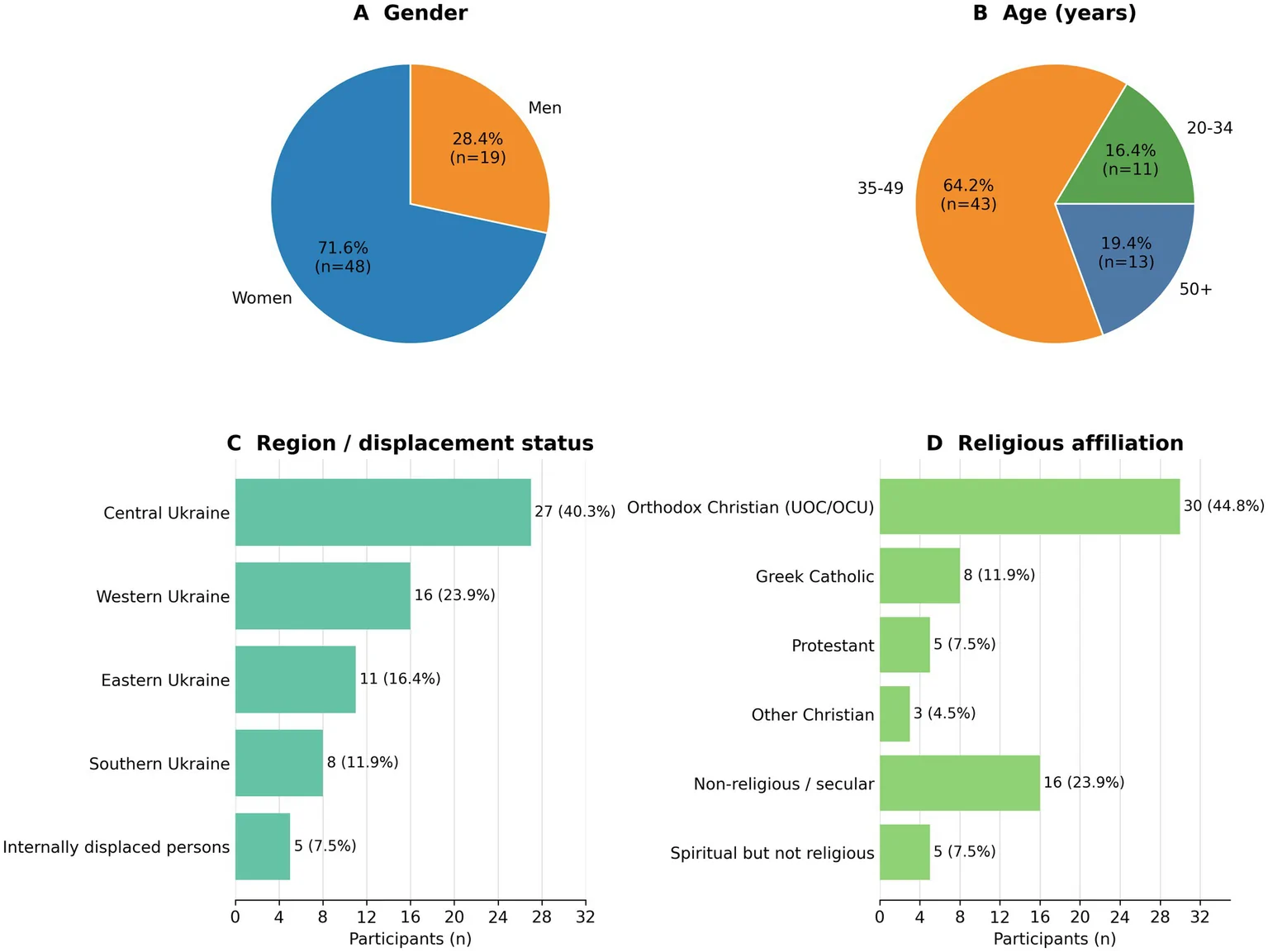
Characteristics of the interview participants (*N* = 67): **(A)** gender; **(B)** age; **(C)** region and displacement status; and **(D)** religious affiliation. Values are shown as participant counts (*n*) and percentages.

Others included Greek Catholics (12%), Protestants (7.5%), other Christian denominations (4.5%), as well as non-religious/secular (24%) and “spiritual but not religious” individuals (7.5%). This variety enables examination of how different belief systems influence coping mechanisms. The professional, geographic, and spiritual diversity of the sample offers a strong foundation for nuanced analysis of cultural coping in wartime. Predominantly female, middle-aged participants, alongside male respondents from various professional backgrounds provide complementary insights into both individual and community resilience.

The study adopted a constructivist epistemology, recognizing that coping strategies are socially and culturally constructed, shaped by historical memory, local value systems, and collective narratives. In this regard, the researchers sought not only to catalogue coping behaviors but to understand their cultural meanings and existential significance. Reflexivity was maintained throughout the research process, with the investigators acknowledging their own cultural and academic positioning in relation to the participants’ lived experiences.

Ethical approval for the investigation was granted by the Swedish Ethical Review Authority (Reference number 2015/126). This particular study also received approval from the Ethics Committee at the University of Gävle and State University “Kyiv Aviation Institute” (Scientific and Technical Council Certificate of Compliance with Research Ethics and Policy in Scientific Research; Date: March 8, 2025; Code: 12/177.1).

The study adhered to fundamental ethical principles, including informed consent, confidentiality, anonymity, and the responsible handling of data. Prior to participation, all respondents received detailed information about the aims of the study, the voluntary nature of participation, confidentiality procedures, and their right to withdraw from the study at any time without consequences. Informed consent was obtained from all participants prior to the interviews. Consent was provided either verbally or through an electronic consent procedure before participation. No signed written consent forms were collected, as the study involved minimal risk, voluntary participation, and anonymous processing of data. These consent procedures were reviewed and approved by the relevant ethics committees and were conducted in accordance with applicable ethical standards for qualitative social science research.

## Results

The analysis generated five themes describing the principal forms of secular existential coping reported by participants: (1) nature and physical space as sources of existential grounding; (2) cultural and creative practices; (3) civic engagement and solidarity; (4) routine, work, and practical activity; and (5) physical activity, meditation, and non-religious contemplative practices. The themes were constructed from the meanings participants attributed to these practices rather than from their formal religious self-identification. Religious and spiritual references occasionally appeared within otherwise secular narratives. Such accounts were treated as hybrid or boundary cases and were not used as primary evidence for defining a secular theme. Their inclusion below serves to demonstrate the empirical permeability of the categories and to prevent an artificial representation of participants’ coping experiences as internally uniform.

### Nature and physical space as sources of existential grounding

The experiences of the respondents highlight the vital role of nature and solitude as secular existential resources during the war. Consistent with psychological research on nature-based coping (Kaplan and Kaplan, 1989, pp. 308–39; Ulrich, 1993, pp. 115–117), natural environments were described as facilitating emotional regulation, attentional restoration, and reflective self-processing. In the Ukrainian context, however, nature also appeared to serve as a bridge to cultural memory and embodied continuity, particularly among individuals with rural roots or ancestral connections to the land. These settings offered a restorative space for sensory and cognitive engagement, supporting recovery without invoking religious or supernatural interpretations.

Solitude similarly emerged across interviews as a meaningful and constructive experience. Respondents consistently described time alone not as a spiritual retreat but as a secular practice of introspection and emotional realignment. For many, solitude functioned as an existential pause—a cognitive holding space enabling reflection, value reassessment, and emotional resilience amid wartime disorientation. A 49-year-old linguist (ID 24) explained, “*Visualization helped me work through problems, and quiet reflection allowed me to reassess my values*.” A 57-year-old university lecturer (ID 38) noted that solitude helped her gather her thoughts, though her primary focus remained on her child’s future and the future of Ukraine. Similarly, a 45-year-old retired woman (ID 6) emphasized, “*Solitude and time in nature were especially helpful, and I came to see how important positive thinking is*.” These accounts portray solitude as an intentional, grounding mental state, fostering clarity and inner strength. Importantly, solitude in these narratives was not equated with loneliness or social withdrawal. Instead, it reflected an autonomous and purposeful mode of coping that aligns with self-determination theory (Deci and Ryan, 2000), which emphasizes the role of autonomy in emotional regulation and identity integration. This secular form of existential coping resonates with Ukraine’s cultural traditions that valorize endurance, reflection, and inner fortitude (Bekh, 2018, p. 184). In line with Frankl’s (2006, p. 45, 61) concept of the “search for meaning,” participants reconstructed significance in their lives not through divine intervention, but through self-directed insight. Thus, both nature and solitude served as culturally embedded, non-religious sources of existential grounding and meaning-making amid the chaos of war.

### Cultural and creative practices

In the Ukrainian cultural context, secular coping during wartime was deeply anchored in cultural expression, creative work, and pragmatic action. National folklore, proverbs, and traditional arts have long emphasized perseverance, diligence, and moral strength in the face of adversity. Expressions such as “Труд перемагає все” (“Labor conquers all”) encapsulate an ethos of secular endurance, functioning not merely as linguistic wisdom but as generational coping frameworks, particularly salient in times of war, famine, or political repression. Traditional arts, especially embroidery (vyshyvanka), weaving, and folk singing have historically operated as non-institutional channels for resilience and identity continuity. Even when stripped of religious symbolism, these practices retain encoded meanings of protection, endurance, and cultural strength (Kushnarenko et al., 2023). For instance, the revival of the patriotic song “Ой у лузі червона калина” (“Oh, the Red Viburnum in the Meadow”) in 2022 became a symbol of secular resistance and collective motivation. Its widespread resonance illustrated how national memory, folk heritage, and aesthetic continuity can ground emotional resilience without invoking metaphysical narratives.

Respondents frequently described turning to creative practices, such as embroidery, drawing, literature, and art therapy—not only as distraction but as purposeful acts of self-regulation and meaning-making. These activities fostered emotional grounding, restored a sense of agency, and sustained cultural coherence amid dislocation and uncertainty. For example, a 49-year-old psychotherapist (ID 18) described using art therapy, gardening, and visualization to cope, explicitly rejecting spiritual or religious frameworks. Similarly, a retired lecturer (ID 19) emphasized the stabilizing role of literature and academic dialogue, while others relied on embroidery, hobbies, or humor to manage emotional states. Even those who identified as religious often reported that creative expression played a more central role in their coping than prayer or religious rituals. A 40-year-old lecturer (ID 7) stated that drawing was more important to her than organized spirituality or physical activity. Similarly, a 53-year-old atheist artist (ID 62) emphasized rest, physical activity, and participation in cultural events rather than introspection or spiritual seeking. These diverse accounts underscore that creativity, cognitive engagement, and cultural practices functioned as secular rituals rooted in memory, embodiment, and expressive autonomy.

Such activities also served as continuity links to Ukraine’s historical traditions of resilience through craft. Embroidery, in particular, was not only an aesthetic outlet but a symbolic act of survival, often practiced by women during past periods of war and exile (Sokolenko, 2022, p. 37; Melikhova, 2023). By engaging in these forms of expression, individuals reclaimed intergenerational narratives of endurance, reinforcing personal coherence and psychological stability in chaotic times. This creative coping, although secular in nature, carried profound existential weight, illustrating how artistic work can affirm life, convey meaning, and preserve identity without recourse to spiritual institutions. As one respondent (ID 29) summarized: “*I maintain my balance through belief and personal creativity*.”

Ultimately, these narratives reflect a multifaceted secular coping landscape, shaped by action, imagination, cognitive reflection, and cultural continuity. Far from being devoid of spirit or depth, secular strategies allowed individuals to construct meaning through artistic agency. In Ukraine, where cultural expression is historically intertwined with national identity and communal survival, these forms of secular meaning-making underscore that resilience need not rely on religious belief. Instead, it can emerge from the creative assertion of values, memories, and inner strength embedded in lived cultural practices.

### Civic engagement and solidarity

Civic engagement and mutual support emerged as secular existential resources when participants described volunteering, helping others, and contributing to their communities as ways of restoring agency, usefulness, moral continuity, and social belonging. Under wartime conditions, such actions enabled participants to move from the passive position of being exposed to danger toward an active position in which they could influence at least part of their social environment. Civic solidarity therefore functioned not only as practical assistance to others but also as a mechanism through which participants reaffirmed personal values and maintained a coherent sense of self.

A small number of participants combined these action-oriented strategies with explicitly religious resources. For example, participant ID 2 described volunteer activity alongside prayer and spiritual mentorship and interpreted her experience through a divinely ordered worldview. Participants ID 14 and ID 16 similarly combined personal effort and community-oriented activity with prayer or church attendance. These accounts were treated as hybrid or boundary cases rather than as representative examples of purely secular coping. The explicitly religious components were not used to define the present theme; analytically, attention was given to the independently articulated role of volunteerism, mutual responsibility, practical action, and social connection.

These hybrid cases qualify, rather than invalidate, the distinction between secular and religious coping. They demonstrate that individuals may draw on several meaning systems simultaneously and that the same outward practice can have different existential meanings. Volunteerism may constitute religious coping when understood as service to God, secular coping when grounded in civic responsibility and human solidarity, or hybrid coping when both interpretations coexist. What distinguishes the secular dimension is therefore not the activity itself but the non-transcendent source of meaning and efficacy attributed to it.

### Routine, work, and practical activity

In the context of wartime conditions, one of the key dimensions of secular coping was the deliberate effort to maintain structure in daily life and to focus on work as a resource for psychological stabilization. In contrast to religious or spiritual interpretations of reality, respondents articulated coping strategies grounded in personal responsibility, agency, and the rational organization of one’s existence. Work, daily responsibilities, the formulation of short-term goals, and adherence to established routines emerged as tools for preserving a sense of subjectivity and emotional balance.

Interview analysis reveals that, for a substantial portion of participants, coping was inextricably linked to practical activity. A 36-year-old respondent explicitly stated that he avoided religious appeals and instead concentrated on his job and achievable daily objectives, which helped him remain grounded. Similarly, a 62-year-old atheist artist described his strategy as entirely centered on routine tasks: work, rest, and the completion of specific duties, devoid of symbolic or metaphysical meaning.

For many women, particularly those in the education sector, coping involved not only the fulfillment of professional responsibilities but also other structured activities, such as caring for pets, engaging in physical exercise, or intellectual self-realization through podcasts or participation in webinars. These activities functioned as a cognitive scaffold, helping to preserve the integrity of the self amid existential threat. It is noteworthy that even among respondents who identified with a general spiritual orientation, a task-oriented coping model prevailed. Statements such as “*I believe in the power of action but surrender the outcomes to a higher force*” (ID 32) reflect a rational integration of philosophical belief with material engagement, wherein the latter occupies a dominant role.

A 45-year-old woman (ID 47), working in humanitarian demining, also expressed doubt: “*Religion did not play a role in how I managed, I found myself questioning the idea of divine justice, especially in light of so much widespread suffering*.” These cases underscore how secular coping may stem not only from neutrality but also from active rejection of religious narratives. A 30-year-old mother employed in explosive risk awareness (ID 57) rejected organized religion altogether: “*Religious platitudes were unhelpful. I’ve long rejected organized religion and prefer to see myself as part of the universe. I also have a deep mistrust of pastoral figures.*” According to the interview, her coping combined therapy, breathing techniques, physical labor, and a broad spiritual sensibility unanchored to religious doctrine.

The accounts of participants ID 32 and ID 57 were treated as hybrid cases because pragmatic, action-oriented coping coexisted with references to a higher force, the universe, or non-institutional spirituality. They were retained in the analysis to demonstrate the complexity of participants’ meaning systems. However, only the independently expressed reliance on work, therapy, breathing techniques, physical activity, and personal responsibility contributed to the construction of the secular theme.

These narratives demonstrate that physical activity ranging from structured exercise to everyday chores was not merely a stress-regulation technique, but often served as a culturally embedded form of meaning-making. In the Ukrainian context, where physical labor has long been culturally valued as a symbol of strength, perseverance, and dignity (Reznikova, 2024), bodily activity took on existential significance. It enabled individuals to reclaim control over their disrupted lives through actions rooted in heritage and habit.

Thus, a structured daily life centered around work emerged not only as an adaptive behavioral strategy but also as a distinct form of existential meaning-making. Participants utilized everyday actions to restore inner order and mitigate disorientation, demonstrating a culturally embedded inclination toward self-regulation and autonomous coping in times of crisis.

### Physical activity, meditation, and non-religious contemplative practices

Physical activities and meditative routines emerged as central forms of secular coping among respondents, offering both psychological stabilization and embodied resilience in wartime conditions. Practices such as yoga, running, walking, and meditative solitude were frequently described as ways to regulate stress, regain control over one’s body, and restore internal balance. For many, these activities served not only as self-care techniques but also as moments of inner grounding that replaced or supplemented traditional religious rituals. A juvenile prevention inspector (ID 46), for instance, emphasized wellness and emotional recovery through regular exercise and meditation, deliberately distinguishing these practices from any religious affiliation. Similarly, several participants highlighted the therapeutic effects of physical labor, gardening, and interaction with nature—activities perceived as stabilizing, purposeful, and conducive to maintaining a sense of continuity amid the chaos of war.

Most participants framed physical activity, meditation, and contemplative routines in embodied and non-transcendent terms, emphasizing stress regulation, bodily control, concentration, and emotional balance. A smaller number referred to universal energy, inner spirituality, or a “Higher Mind.” These latter accounts were classified as spiritual or hybrid rather than as purely secular. They were retained as boundary cases, while their physical or meditative practices contributed to the secular theme only when participants independently attributed their effectiveness to bodily awareness, self-directed attention, or emotional regulation rather than to transcendent intervention.

Conversely, others demonstrated an explicit rejection of religious interpretations of suffering, instead embracing pragmatic and experiential approaches to coping. Even when participants expressed skepticism or existential doubt, they engaged in physical and meditative routines—walking, sports, or mindfulness exercises—that provided emotional regulation and existential anchoring.

Within Ukrainian cultural traditions, physical engagement with the natural or domestic environment—such as gardening, working in the yard, or maintaining household routines—carries deep symbolic meaning, associated with endurance, moral integrity, and self-reliance. As Bekh (2018) notes, such practices constitute a “pedagogy of endurance,” where disciplined physical effort embodies a culturally ingrained mechanism of resilience.

Overall, physical activity and non-religious contemplative practices functioned as predominantly secular embodied coping strategies that integrated bodily regulation, self-directed attention, and culturally meaningful routines. In a limited number of hybrid cases, these practices coexisted with individualized spirituality. This variation further demonstrates that secular and spiritual coping should be understood as analytically distinguishable dimensions that may nevertheless coexist within a single participant’s narrative.

## Discussion

The findings of this study highlight the diverse and culturally grounded ways in which Ukrainian civilians have coped with the psychological and existential challenges posed by the full-scale war. While religious coping has often been foregrounded in trauma studies conducted in contexts where faith plays a prominent societal role, this research draws attention to secular existential strategies that have been central to many participants’ meaning-making processes.

Participants frequently relied on culturally embedded forms of expression—such as embroidery, creative work, and participation in cultural events—not merely as distractions, but as deeply symbolic acts of continuity and identity preservation. These practices serve as what Ahmadi (2006) refers to as “meaning-making coping strategies”: culturally and morally grounded responses to trauma that allow individuals to re-establish coherence in disrupted life narratives. In this study, such strategies were often enacted without reference to religion, indicating a broader existential orientation rooted in tradition, values, aesthetics, and the human connection to nature.

The prominence of nature and solitude in respondents’ coping highlights the embodied and sensory dimensions of resilience. These practices align with earlier findings on nature-based coping in non-religious populations, where the physical environment functions as both a source of stability and a metaphoric space for reflection (Ahmadi and Ahmadi, 2015; Ahmadi et al., 2018). In war-torn environments, where public spaces are often destroyed or inaccessible, nature becomes a private sanctuary for emotional regulation and existential grounding.

Civic engagement and mutual aid also emerged as significant pathways to resilience. These findings echo the growing body of research on collective coping in humanitarian crises (Masten, 2014; Zandi and Ahmadi, 2024), suggesting that moral action and community solidarity not only benefit others but serve as powerful self-regulatory mechanisms. Participants who engaged in volunteering or neighbor support often described these activities as means of restoring agency and dignity in a context otherwise marked by helplessness and loss.

Considered across the five themes, the central analytical pattern was not the presence of any single coping activity but the reconstruction of agency, continuity, and relational embeddedness. Nature and solitude created continuity between the pre-war and wartime self; creative practices connected personal experience with cultural memory; work and routine restored temporal structure; embodied practices re-established a sense of control; and civic solidarity transformed individual vulnerability into socially recognized action. These practices became existentially significant because they allowed participants to experience themselves as acting subjects rather than solely as recipients of violence and disruption.

The hybrid and deviant cases further refine this interpretation. Some participants combined secular practices with prayer, religious participation, or non-institutional spirituality, whereas others explicitly rejected religious explanations because of wartime suffering. These contrasting accounts indicate that secular coping is not simply equivalent to atheism or the absence of religion. Rather, it refers to the source from which a particular practice derives its meaning and perceived efficacy. The same activity may be experienced as secular, spiritual, or religious depending on whether participants attribute its effectiveness to personal agency and social connection, to a transcendent force, or to an institutionalized faith tradition. This interpretive distinction explains why participants with a religious identity could still rely predominantly on secular existential resources.

Notably, the secular orientation of many coping strategies in this study reflects a shift in the psychological landscape of post-Soviet societies. While religion remains a valued source of support for many, there is increasing evidence of pluralism in meaning-making, including reliance on internal values, cultural memory, and personal philosophies. This evolution calls for a broader conceptualization of coping that includes secular existential practices alongside religious and clinical models.

Theoretical perspectives such as the BASIC Ph model (Lahad, 2016) and the meaning-making framework developed by Ahmadi (2006) help contextualize these findings within a multidimensional understanding of trauma response. The Ukrainian participants in this study employed coping tools across cognitive, behavioral, emotional, and symbolic domains. Importantly, these were not fixed strategies but dynamically adapted to ongoing changes in the war context—highlighting the need for flexible and culturally informed mental health interventions.

Furthermore, the coping responses observed in this study appear deeply informed by Ukraine’s historical experiences with collective trauma—from the Holodomor and Soviet repression to the Chernobyl disaster and the 2014 war. These layers of historical memory have contributed to a cultural schema of endurance that informs present-daycoping. Rather than pathology, many responses represented adaptive efforts to affirm life, restore meaning, and uphold dignity amid violence and uncertainty.

## Conclusion

This study contributes to a deeper understanding of how secular existential coping strategies function in the context of armed conflict. The qualitative data gathered from Ukrainian civilians illustrate the central role of culturally embedded, non-religious meaning-making in times of extreme stress and uncertainty. Rather than relying solely on clinical or religious frameworks, many individuals turned to nature, creative expression, civic solidarity, structured routine, and personal reflection as pathways to psychological endurance and existential coherence.

The findings underscore that secular coping is not the absence of meaning, but rather a different form of meaning-making—one that draws from local cultural heritage, historical memory, moral values, and relational connectedness. These strategies reflect a dynamic process in which individuals construct and reconstruct meaning in response to traumatic events, often blending personal, philosophical, and communal resources. By highlighting the plurality of coping mechanisms among Ukrainians, this study challenges narrow definitions of resilience that privilege clinical or religious models. It calls for more inclusive approaches to trauma intervention that recognize the cultural, existential, and non-institutional dimensions of human adaptation.

Future research should explore how these secular coping practices evolve over time and how they intersect with religious, spiritual, and professional forms of support. Furthermore, mental health interventions in conflict settings should consider integrating culturally relevant, non-religious tools for meaning-making and psychological support. In recognizing the cultural depth and existential agency embedded in everyday acts of survival, this study affirms the resilience of the Ukrainian people and contributes to broader efforts to develop context-sensitive models of psychosocial care in wartime.

### Limitations of the study

Several limitations should be considered. First, the sample was predominantly female and middle-aged, and therefore does not represent all demographic groups affected by the war. Second, the findings are based on self-reported narratives and may be influenced by memory, subjective interpretation, and participants’ willingness to discuss emotionally sensitive experiences. Third, the boundaries between secular, spiritual, and religious coping are analytically useful but not always clear-cut in lived experience. Several participants drew on more than one meaning system, and the classification of hybrid accounts necessarily involved interpretive judgment. The study therefore does not seek to establish the prevalence of particular coping strategies or to assign participants to fixed belief categories. Finally, the cross-sectional design captures coping at a particular stage of the ongoing war; longitudinal research is needed to examine how these strategies and their meanings change over time.

## Data Availability

The datasets generated and analysed for this study are not publicly available because they contain potentially identifiable qualitative interview data. Further information may be made available by the corresponding author upon reasonable request, subject to ethical approval and applicable data-protection requirements.
